# Synthesis of Secretory Proteins in *Yarrowia lipolytica*: Effect of Combined Stress Factors and Metabolic Load

**DOI:** 10.3390/ijms23073602

**Published:** 2022-03-25

**Authors:** Maria Gorczyca, Jan Kaźmierczak, Patrick Fickers, Ewelina Celińska

**Affiliations:** 1 Department of Biotechnology and Food Microbiology, Poznań University of Life Sciences, 60-637 Poznań, Poland; maria.gorczyca@up.poznan.pl (M.G.); jan.kazmierczak96@gmail.com (J.K.); 2 Microbial Processes and Interactions, TERRA Teaching and Research Centre, Gembloux Agro-Bio Tech, University of Liege, 5030 Gembloux, Belgium

**Keywords:** heterologous protein, secretion, yeast, aeration, pH, stress, metabolic burden

## Abstract

While overproduction of recombinant secretory proteins (rs-Prots) triggers multiple changes in the physiology of the producer cell, exposure to suboptimal growth conditions may further increase that biological response. The environmental conditions may modulate the efficiency of both the rs-Prot gene transcription and translation but also the polypeptide folding. Insights into responses elicited by different environmental stresses on the rs-Prots synthesis and host yeast physiology might contribute to a better understanding of fundamental biology processes, thus providing some clues to further optimise bioprocesses. Herein, a series of batch cultivations of *Yarrowia lipolytica* strains differentially metabolically burdened by the rs-Prots overproduction have been conducted. Combinations of different stress factors, namely pH (3/7) and oxygen availability (kLa 28/110 h^−1^), have been considered for their impact on cell growth and morphology, substrate consumption, metabolic activity, genes expression, and secretion of the rs-Prots. Amongst others, our data demonstrate that a highly metabolically burdened cell has a higher demand for the carbon source, although presenting a compromised cell growth. Moreover, the observed decrease in rs-Prot production under adverse environmental conditions rather results from the emergence of a less-producing cell subpopulation than from the decrease of the synthetic capacity of the whole cell population.

## 1. Introduction

It is well recognized that high-level synthesis of recombinant secretory proteins (rs-Prot) has a direct impact on the host cell metabolism (also known as metabolic burden), often negatively affecting biological parameters such as growth rate, biomass yield, and specific substrate consumption rate [1,2,3,4,5]. Indeed, recombinant gene expression leads to an increase in transcription and translation, which may become limiting at very high levels due to depletion of precursors (nucleotides, amino acids) and energy [6]. Depending on the biochemical characteristics of the rs-Prots considered (size, type of posttranslational modifications), the intensity of the metabolic burden may vary, with the consequence that some biological processes may become bottle-necked [4,5]. On top of that, during the rs-Prots production process the host cell is exposed to various environmental factors that awake specific biological responses to maintain homeostasis. In this sense, the rs-Prot producer cell is subjected to intrinsic and extrinsic stress factors. 

Amongst the common yeast expression hosts used for rs-Prots synthesis, like *Komagataella phaffii* or *Saccharomyces cerevisiae* [7], *Yarrowia lipolytica* has gained significant attention. It is a nonconventional yeast species, known for its high capacity in accumulate intracellular lipids, and the ability to grow on a wide variety of substrates, including carbohydrates, alcohols, acids organic, and hydrophobic compounds (e.g., alkanes, fatty acids, and triglycerides) [8,9,10]. *Y. lipolytica* is also characterised by several physiological traits that are specifically advantageous for overproduction of rs-Prots and thus for industrial exploitation [11,12]. Its robust secretory pathway, resembling filamentous fungi-like system [13], is particularly useful in this regard, as the secretion of rs-Prots offers multiple advantages over their retention inside the producer cells, like ease of purification, higher yields, and full maturation of the polypeptide including post-translational modifications. *Y. lipolytica* nominal growth ability spans over a wide range of pH, temperature and osmolarity values [14,15], highlighting thus its robustness in term of metabolism. The optimum pH for typical representatives of *Y. lipolytica* is close to pH 5–5.5. Only a few, specific isolates can tolerate very low (2.0) or even very high pH values (9.7) [14,15,16]. For strain W29, the growth ability at pH 3 and 7 is about 75% of that obtained at the optimal value (pH 5) [17]. As obligate respiratory organism, a critical factor that is technically challenging in *Y. lipolytica*-based rs-Prots production process is oxygen availability (OA) [18,19,20]. Sufficient supply of oxygen was found to be the key factor affecting production capacity of multiple target products (enzymes and metabolites) by *Y. lipolytica*, precluding high efficiency of the corresponding process [21,22,23,24,25]. We recently reported that a volumetric oxygen transfer coefficient (kLa) lower than 50 h^−1^ (i.e., low OA) negatively affects cell growth and carbon source uptake rate as compared to values above 100 h^−1^ (high OA) [19].

The effect of various environmental conditions on the synthesis of native and recombinant proteins in *Y. lipolytica* has been addressed in several previous works. With the aim to study the impact of local gradients in pH and OA encountered in large-scale bioreactor, a scaled-down approach was used to investigate individual responses awakened by limited OA and fluctuations in pH, implemented independently [22]. It was found that insufficient OA had a pronounced effect on the gene expression and ultimately on the synthesis of the corresponding rs-Prot, while the simulated pH gradient was of less significant impact. In our previous study, we observed that low OA limits rs-Prot synthesis, starting from the transcription stage, and that the limited rs-Prot formation is not related to cell growth ability [19]. We also evidenced the positive effect of decreased growth temperature [26] and the adverse impact of increased osmolarity [27] on rs-Prots production in *Y. lipolytica*. Nevertheless, the combination of several extrinsic stress factors and their effect on differently metabolically burdened cells has not been studied to date.

In this work, we sought the combined impact of intrinsic and extrinsic factors on *Y. lipolytica* cell physiology and the ability to synthesize rs-Prot. The experimental setup combined three variables, two physico-chemical parameters, namely pH and OA, and the type of rs-Prot as a biological parameter (i.e., size, complexity of posttranslational modifications). Based on data reported in the literature [15,17,28,29], pH of 3 and 7 were selected as stressing conditions (low and high values as compared to the optimal pH 5), while kLa of 28 h^−1^ and 110 h^−1^ were used as low and high OA conditions according to our previous study [19]. Two *Y. lipolytica* strains significantly differed in terms of metabolic load caused by overexpression of a single gene encoding either small (27 kDa), negligibly post-translationally modified protein (YFP), or two medium sized proteins (52 kDa and 65 kDa), of which one is highly disulphide-bonded (alpha-amylase; SoA) and the other is highly glycosylated (glucoamylase; TlG). The molecular landscape, awakened by the overexpression of those genes, has recently been characterised by global transcriptome profiling [5], and the obtained results evidenced that overproduction of YFP and TlG/SoA triggers a “low” and “high” metabolic burden to the host cells, respectively. Herein, these strains were cultured under a combination of the selected environmental stress conditions and characterised in terms of growth, substrate consumption, metabolic activity, morphology, gene expression and recombinant protein secretion.

## 2. Results

### 2.1. Cell Growth and Substrate Consumption Kinetics by the Differentially Burdened Strains under Extrinsic Stress Conditions

**Figure 1:**
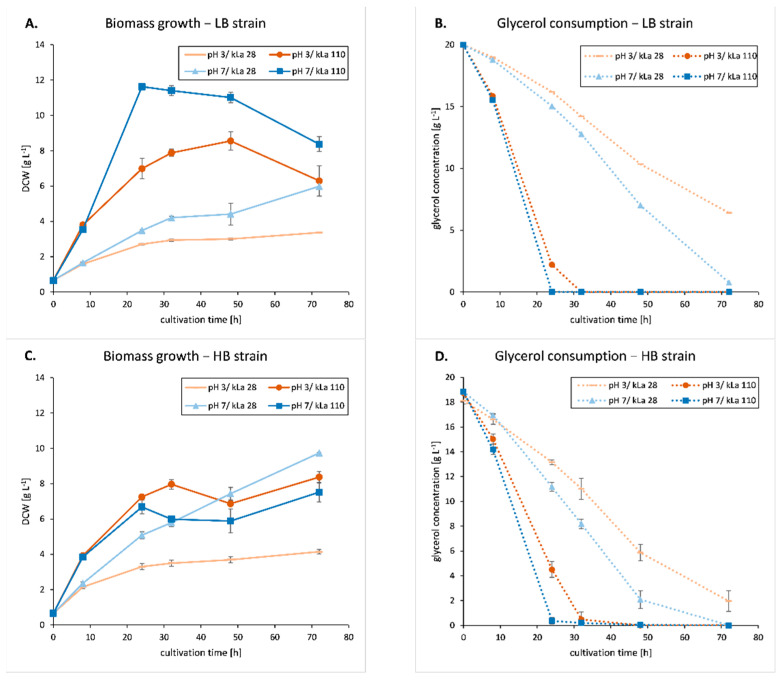
Biomass growth (**A**,**C**) and glycerol consumption (**B**,**D**) by *Y. lipolytica* strains overproducing YFP (LB strain—**A**,**B**) or TlG+SoA GGY178 (HB strain—**C**,**D**) rs-Prots. Mean values of biological triplicate ±SD are shown.

The implemented culturing conditions, ranked from the most advantageous (pH 7, kLa of 110 h^−1^) to the least beneficial (pH 3, kLa of 28 h^−1^) ones, had a significant impact on the low (LB) and high (HB) metabolically burdened strains in terms of cell growth and substrate consumption (Figure 1, Table 1 and Figure S1 for the non-burdened control strain). 

For pH 7 and kLa 110 h^−1^, the highest biomass value was obtained after 24 h for the YFP-producing strain (11.6 gDCW L^−1^) while the HB strain yielded to a maximal value of 7.5 gDCW L^−1^ (Figure 1A,C, Table 1; different at *p* < 0.05). Its growth rate, Q_gDCW_, was also significantly lower as compared to the LB strain (Table 1; *p* < 0.05). The differences between the two strains were substantially less marked in terms of maximal biomass and growth rate for the other culture conditions tested. Considering the pH as the variable factor at a fixed OA, the acidic environment caused a significant decrease in growth rate for the LB strain at high OA (Figure 1A,B, Table 1, *p* < 0.05). On the other hand, for the HB strain, the downshift to pH 3 caused a significant decrease in growth rate under low OA (*p* < 0.05) but had no marked impact under high OA (Table 1). Finally, a similar limiting impact of low OA on cell growth was observed (*p* < 0.05). At pH 3, significant differences (i.e., 20%) in growth rate (0.09 and 0.11 gDCW L^−1^ h^−1^) and tendencies in biomass growth (3.4 and 4.1 gDCW L^−1^) were observed for LB and HB strains, respectively. Under pH 7 and low OA, the differences in growth rate (0.12 and 0.18 gDCW L^−1^ h^−1^) and biomass (6 and 9.7 gDCW L^−1^) between the two strains were clearly marked (*p* < 0.05; Table 1). In these conditions, the growth rate of the HB strain was 33% higher than that of the LB strain. For strain LB, the growth kinetics were similar to that of the control strain (i.e., not metabolically burdened) for all the culture conditions tested, confirming thus that YFP synthesis does not induce a significant metabolic overload, by contrast to TIG and SoA synthesis.

For the LB strain, glycerol consumption was positively correlated with the biomass values (r = 0.875 across consecutive time-points; Figure 1A,B, Table 1). This was not the case for HB strain, as the highest biomass (9.7 gDCW L^−1^, pH 7/kLa 28 h^−1^) was obtained for a low value of glycerol consumption rate (0.37 g L^−1^ h^−1^). On the other hand, the highest glycerol consumption rate (0.82 g L^−1^ h^−1^, pH 7/kLa 110 h^−1^) led to a low value of biomass (7.5 gDCW L^−1^) for this strain (Figure 1C,D). For the HB strain, the reduction of growth observed for culture condition pH 7/kLa 110 h^−1^ (Figure 1A,C, Table 1) was not accompanied by a reduced glycerol consumption rate (Table 1). Moreover, glycerol uptake rates were similar for the two strains under these conditions (0.83 vs. 0.82 g L^−1^ h^−1^; Table 1), which suggests a high metabolic activity for both strains. However, the biomass yield (Yx/s) was 1.7-fold lower for HB strain as compared to LB strain (0.55 vs. 0.31 g g^−1^, respectively). Biomass yield was significantly higher (at *p* < 0.05) for the LB strains as compared to the HB strains, cultured under the corresponding conditions (Table 1). At the same time, the Q_gly_ parameter was equal or higher for the HB as compared to LB strain, except for the pH 3/kLa 110 h^−1^ (Table 1). This suggests a redirection of metabolic fluxes from biomass formation to, most likely, the “costly” rs-Prot synthesis or to any energy loss arising from side metabolisms. 

Altogether, these observations highlighted that both OA and metabolic burden caused by rs-Prots overproduction are the two significant factors affecting growth parameters of the tested producer strains, at least under studied experimental conditions. On the other hand, pH has a lower impact on cell growth and substrate consumption rate, with some favourable impact of neutral pH. A combination of low pH and limited OA is particularly detrimental and leads to growth cessation and incomplete consumption of the substrate within the culturing time. Under sufficient provision of oxygen (kLa 110 h^−1^) and neutral pH, the heavily burdened HB strain was subjected to growth limitation, which cannot be explained by limited metabolic activity, as the substrate consumption rate was unaffected. Glycerol uptake was favoured at high OA, and, under these conditions, it was equivalent for both strains. However, under oxygen limitation, glycerol uptake was much higher for the highly burdened strain as compared to the less burdened one. This suggests that a combination of intrinsic and extrinsic stress factors is associated with an increased demand for substrate. 

### 2.2. Heterologous Gene Expression under the Applied Stress Conditions

**Figure 2:**
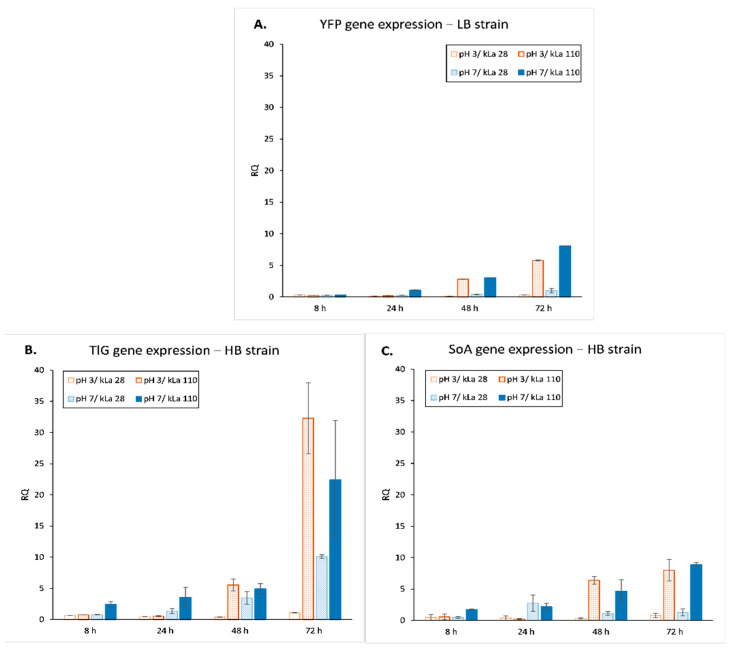
Relative expression level of YFP (**A**), TlG (**B**), and SoA (**C**) gene in *Y. lipolytica* LB strain (**A**) and HB strain (**B**,**C**) throughout culturing under specified conditions. Mean values of biological triplicate ±SD are shown.

Transcripts levels of the three heterologous genes (YFP, SoA, TlG) were determined at different time points over 72 h (Figure 2). As clearly stems from Figure 2, high OA is a prerequisite for high transcriptional activity (i.e., high expression level). Starting from 48 h of culture, the expression levels for all the tested genes under high OA equalled at both pH, highlighting the key impact of sufficient oxygen provision and not pH. Indeed, implementation of high OA triggered an increased expression of the genes by ~2- to 30-fold as compared to their low OA counterparts. Under low OA, cultures conducted at pH 7 led to slightly higher transcriptional activity as compared to pH 3. Overall, these observations highlight that under the adopted culture conditions, sufficient oxygen provision is the key factor impacting efficient transcription of the three heterologous genes. Acidic pH exerted negative impact on the rs-Prots-encoding genes transcription only in combination with low OA.

### 2.3. Metabolic Activity and Morphology of the Cells

**Figure 3:**
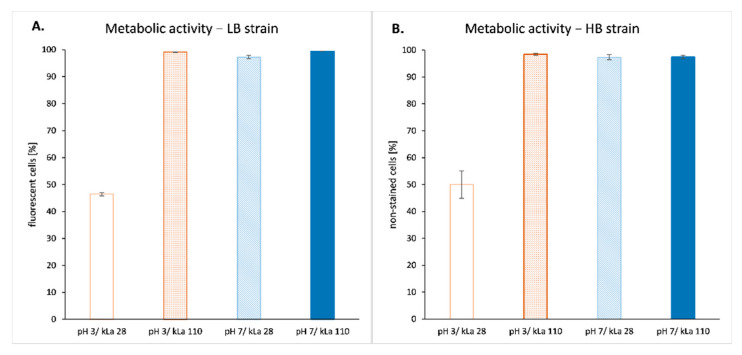
Percentage of metabolically active cells within populations of *Y. lipolytica* LB strain (**A**) and HB strain (**B**) cultured under specified conditions. The metabolic activity of a cell was determined based on intracellular FL (LB strain) or the ability to oxidize methylene blue dye (HB strain) studied in 20,000 or 5000 cells, respectively. Mean values of biological triplicate ±SD are shown.

Metabolic activity of the cells under the different culture conditions tested was assessed either directly or indirectly depending on the strain considered. For HB strain synthetizing the two recombinant enzymes, methylene blue staining and light microscopy were used to discriminate between highly metabolically active and non-active cells. Indeed, methylene blue readily permeates all the yeast cells and is reduced to a colourless compound in metabolically active cells. Less metabolically active cells turn light blue, while non-active cells remain dark blue. For the YFP strain (LB), the median value of intracellular fluorescence measured by flow cytometry was used as a proxy to estimate the cell metabolic activity, according to previous publications [30,31,32]. Cells with median fluorescence value lower than 20,000 FU were considered as non-metabolically active (i.e., not producing YFP) while those with higher value were considered as metabolically active. The threshold of 20,000 FU was defined based on the median fluorescence value obtained with the control strain (YFP-negative). For all the culture conditions tested, only condition pH 3/kLa 28 h^−1^ had a detrimental effect on cell metabolic activity for both LB and HB strains (Figure 3), and no difference was seen between the strains in this regard.

**Figure 4:**
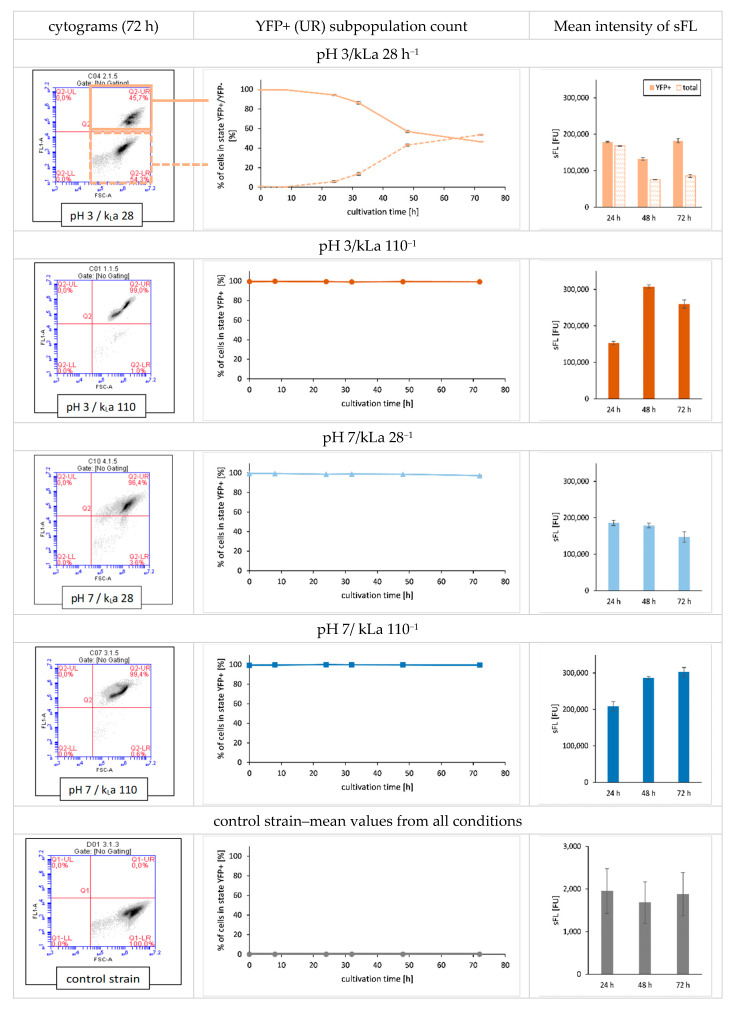
Phenotypic analysis of *Y. lipolytica* LB strain populations cultured under specified conditions in terms of viability and intensity of intracellular FL. Cytograms (**left** panel) show the intensity of FL (*y* axis; FL-A channel) vs. morphology (*x* axis: FSC-A channel) at 72 h of culturing. Kinetic curves (**middle** panel) show percentage of metabolically active cells within the populations throughout the culturing time (*y* axis: %; *x* axis: time [h]). Histograms (**right** panel) show the mean value for intensity of FL in FU at indicated time-points. Cytograms are representative for biological triplicates. Curves and histograms show mean values of biological triplicate ±SD.

As a further characterisation, cellular metabolic activity of LB strain was evaluated over time at a single cell level by flow cytometry. As shown in Figure 4, in pH 3/kLa 28 h^−1^, a fraction of the LB cells switched over time from a metabolically active state (YFP producing phenotype, FU > 20,000; gate Q1-UR in the cytograms) to a non-metabolically active state (FU < 20,000; gate Q2-LR in the cytograms). After 72 h of culture, 53.6% of the cell population were in a non-metabolically active state. The lower mean fluorescence intensity values (Figure 4) for total population observed in those conditions was a consequence of the emergence of the non-metabolically active sub-population (YFP non-producing phenotype) rather than a global decrease of metabolic activity of the whole population. This heterogeneity in the cell population was not observed for the other experimental conditions tested. For condition pH 3/kLa 28 h^−1^, the sub-population of metabolically active cells emitted FL at the same level as that observed for pH 7/kLa 28 h^−1^ (sFL close to 200,000 FU).

**Figure 5:**
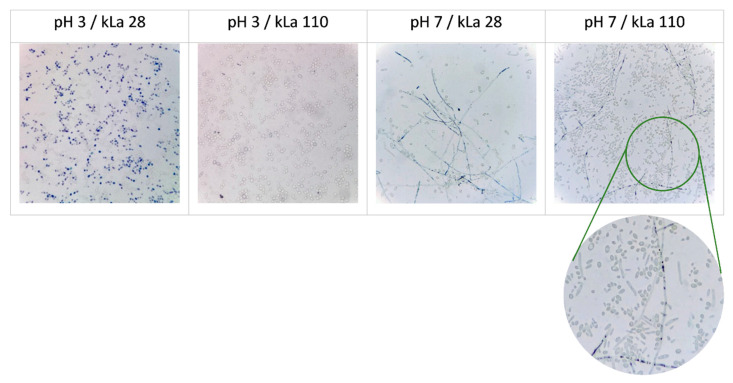
Microscopic images of *Y. lipolytica* HB strain cultured under specified conditions. The preparations were stained with methylene blue and observed under 400× magnification. Images are representative for a larger number of observations.

**Figure 6:**
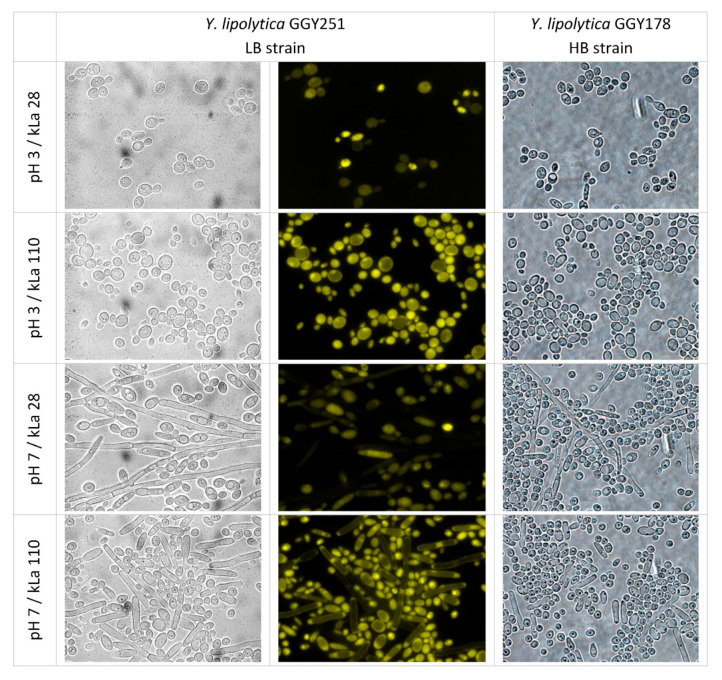
Microscopic images of *Y. lipolytica* LB and HB cells cultured under specified conditions (1 column) observed under 1000× magnification in white light (**left** and **right** panels) and under fluorescent microscope (**central** panels). Pictures are representative for the cell morphology starting from 24 h of culturing. Identical fields are shown in left and central panel. Images are representative for a larger number of observations.

Regarding the cell morphology, the cells adopted an ovoid morphotype at pH 3 irrespective of the OA or strain type (Figure 5 and Figure 6). By contrast, at pH 7, a mix of ovoid cells and filaments was observed, and some relationship between the morphotype and OA could be observed. At pH 7 and low OA, the cells tend to form more filaments which were, at the same time, longer and sometimes branched, while under high OA shorter filaments and more ovoid cells were seen. Interestingly, FL emitted by the longest filaments of LB strain was weaker (visual inspection, Figure 6), but also, most of those very long filaments of the HB strain were intensively stained with methylene blue, suggesting a lower metabolic activity of this morphotype.

### 2.4. Secretion of Active rs-Prots under High and Low OA

**Figure 7:**
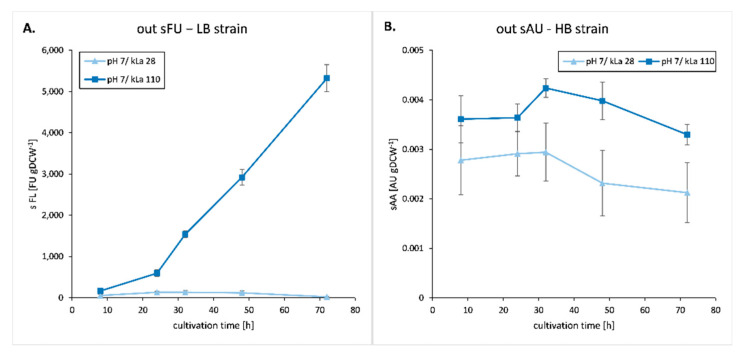

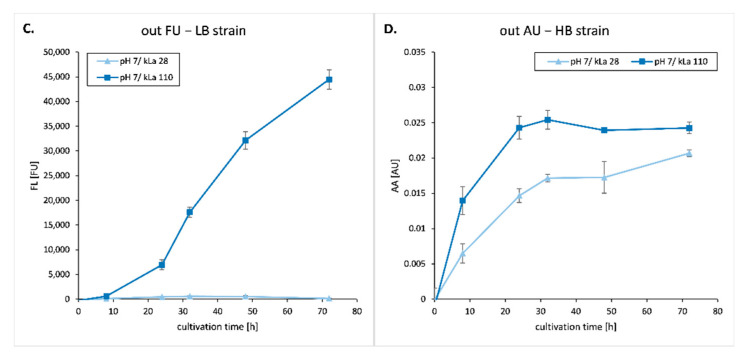
Extracellular level of FL (**A**,**C**) or AA (**B**,**D**) in *Y. lipolytica* LB (**A**,**C**) and HB (**B**,**D**) strain cultures under specified conditions (pH 7). Values are expressed as total reads (**C**,**D**) or specified to biomass (**A**,**B**). Mean values of biological triplicate ±SD are shown.

Specific and total extracellular YFP fluorescence and amylolytic activity in culture supernatant were used as a mean to monitor rs-Prot secretion over time. This was performed only for cultures at pH 7, as at pH 3 both YFP fluorescence and amylolytic activities were impaired, most likely due to protein degradation (data not shown). As shown in Figure 7, specific FU (sFU) and total FU (FU) increased over time at high OA (kLa 110 h^−1^) while they were near to zero under low OA. On the other hand, the specific amylolytic activity (sAA) remained constant over time at high OA while it tended to slowly decrease at low OA (Figure 7b; not a statistically significant decrease).

Specific fluorescence (sFL) and amylolytic activity (sAA), serving as a proxy of protein secretion, were negatively correlated with the culturing time (negative r coefficient values and *p* ≤ 0.05, Figure S4). This in part results from limited but progressing biomass growth (Figure 1) impacting the measures normalized per biomass. Strikingly, under high OA/pH 7 (i.e., the most advantageous conditions), specific FL increased continuously only for the LB strain (YFP), while for the HB strain-sAA normalized per biomass remained at a constant level throughout the culture, mimicking biomass growth (Figure 7B and Figure S4). A continuous increase in specific FL for the LB strain indicates a continuous increase of protein synthesis and secretion, which results from a continuously increasing expression of YFP gene under high OA/pH 7 (Figure 2). By comparison, the lack of such correlations in HB strain under these conditions suggests that the secretory pathway reached its maximum capacity early in the process. Linear increase in the secreted fraction of the rs-Prot occurred solely for the YFP-overproducing strain under the most advantageous environmental conditions (Figure S4b).

### 2.5. Gene Expression vs. rs-Prot Synthesis

**Figure 8:**
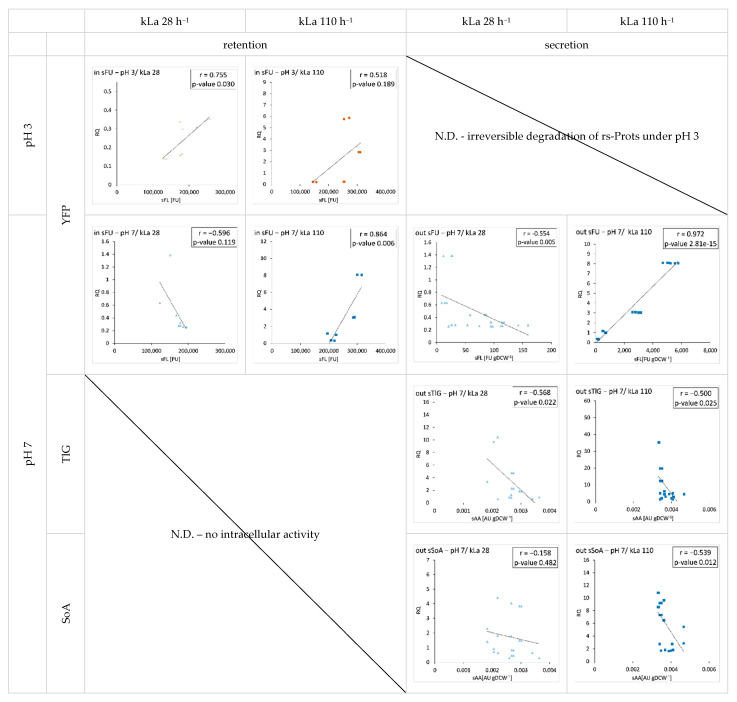
Analysis of correlation between heterologous genes transcript level (*y* axis; RQ) and intracellular or extracellular FL or AA (*x* axis; sFL, sAA) of *Y. lipolytica* LB and HB strains cultured under specified conditions. Culturing conditions are given in the headings of columns and rows, and as sub-figures headings. r values denote Pearson correlation coefficients. N.D.—not determined due to indicated reasons.

With the aim to highlight a significant correlation between transcription level and secretion of the rs-Prots, a Pearson correlation analysis between the two parameters was conducted (Figure 8). 

Significant positive correlation between transcription and extracellular amounts of the reporter proteins would imply no bottlenecks hampering synthesis of the final active rs-Prots. Significant positive correlation of the transcript level and the intracellular (r = 0.864) or the secreted protein fraction (r = 0.972;) was observed solely for the LB strain grown at pH 7 and kLa 110 h^−1^. Oxygen limitation uniformly triggered negative correlation between accumulated transcripts and extracellular amounts of rs-Prots, which can be deduced from negative r coefficients value (Figure 8; significant negative correlation was observed for out_sTlG and out sFU and a tendency for out_sSoA). Interestingly, for all the enzymatic rs-Prots studied here, being medium-sized, post-translationally modified proteins, an increase in transcripts level was accompanied by a decrease in their extracellular amounts, as indicated by significant negative correlation values (Figure 8), except for SoA at pH 7/kLa 28 h^−1^.

It can be concluded that for the LB strain, limited OA caused a loss of positive correlation between transcription and secretion. On the other hand, in case of the HB strain, it can be speculated that the metabolic effort required for overproduction of the two heavily post-translationally modified proteins was so high that even advantageous environmental conditions could not overweight the stress imposed by the rs-Prot synthesis. This is illustrated by the negative values of the Pearson correlation coefficients.

## 3. Discussion

The aim of this research was to investigate the combined impact of the metabolic burden imposed by the overproduction of heterologous secretory proteins and adverse environmental conditions on the physiology of producer cells. To this end, two recombinant *Y. lipolytica* strains that served as models of low (LB) and high (HB) metabolic load were used. The LB strain expresses a gene encoding a small, fluorescent reporter protein (YFP) under a strong, growth-phase dependent promoter. Our recent investigation on the *Y. lipolytica* cell physiology [4] and its molecular background revealed by transcriptomics [5], demonstrated that YFP can be synthesized and secreted at very high levels without imposing metabolic burden or unfolded protein response in the host cell. This was confirmed here by comparison of the growth kinetic obtained for the LB strain as compared to that of the control strain in all the tested culture conditions. We also previously reported that even at low OA (kLa 28h^−1^), oxygen provision is still sufficient to support full maturation of GFP-like fluorophores in *Y. lipolytica* [19]. By contrast to YFP, we highlighted that overproduction of the medium-sized proteins, especially heavily glycosylated TlG, has a significant adverse impact on the producer cell in terms of physiological and molecular responses. Herein, the overexpression of two proteins, namely SoA and TlG, bottlenecking different stages of folding and maturation of polypeptides, further added to the intensity of the imposed stress. The model strains were subjected to a series of batch cultivations with implemented stress factors, namely acidic pH and limited OA, applied alone or in combination. Although *Y. lipolytica* is known to grow well in a wide pH range without relevant impact on growth [17,28,29,33,34], oxygen deficiency is a strong limiting factor for this species [18,19,22,28]. Combination of the extrinsic stress factors and metabolic load in relation to growth, morphology and rs-Prots synthesis has not been studied previously in *Y. lipolytica*. 

Our primary observation was that indeed, depending on the metabolic burden intensity, the cells responded differently to the culture conditions tested. Foremost, we observed an unexpected limitation in growth of the HB strain under high OA (especially pH 7; between 32 and 48 h), which was not accompanied by a lower rate of glycerol consumption (Figure 1). In addition, the growth retardation overlapped with the onset of the heterologous gene overexpression (Figure 2). Based on growth kinetics, substrate consumption rate, and heterologous gene expression, we postulate that the lower growth of HB strain is the result of intrinsic metabolic burden caused by overproduction of the two “highly demanding” proteins. As a side observation, we noticed a continuous increase in the heterologous gene expression and rs-Prots synthesis despite depletion of glycerol (Figure 1 and Figure 2). A similar observation was made for the *Y. lipolytica* strain overexpressing the CalB lipase gene from *Candida antarctica* in batch bioreactor [35]. This can be easily explained by the availability of complex medium components (i.e., peptone) which are known to serve as a sufficient nutrient source to support the growth of *Y. lipolytica* and rs-Prot synthesis [36]. In addition, *Y. lipolytica* is known to produce some typical metabolites (erythritol, mannitol, and α-ketoglutaric acid) at gram per litre, whose re-consumption partly secured the decreasing amount of carbon in the terminal stages of the cultures [37].

Gene expression results (Figure 2) revealed gene-dependent differences in the relative transcripts level under a given culture variant, which could be attributed to variation in the transcription rate, mRNAs stability, and pace of translation. In addition, in the majority of cases, an increase in the transcript quantity was accompanied by a decrease in the extracellular amounts of the corresponding protein (Figure 8). Interestingly, similar results were obtained previously [3], showing almost a 3-fold higher transcript level for a gene encoding a larger protein (120 kDa) as compared to that encoding a smaller protein (55 kDa), when both were cloned under the same promoter and at defined genomic locus. However, the amount of active, secreted protein was 190-fold lower than the former when compared to the latter. The authors suggested that the lower secretion efficiency could result from an overload of the secretory pathway by the “large protein” and their targeting into endoplasmic reticulum associated degradation (ERAD). The other explanation proposed by the authors, could be an insufficient translation capacity, leading to accumulation of transcripts and their subsequent degradation. It is highly plausible that such a mechanism also took place in the HB strain, especially considering negative correlations between the transcript accumulation levels and secretion of TlG and SoA proteins under all conditions. Our recent molecular insight into these phenomena suggests that both explanations proposed by [3] could be valid. We previously observed that the overexpression of a gene encoding highly glycosylated protein (TlG) induced vacuolar protein trafficking and degradation [5], while overexpression of a gene under unfavourable conditions led to translation limitation by concerted decrease in tRNA aminoacylation reaction [27]. On the other hand, our current observations (Figure 4) suggest that the observed changes in the transcripts level, measured globally for a whole cell population, may result from a phenotypic heterogeneity of the producer cell population, induced by unfavourable conditions.

Although the heterologous gene expression levels were different for each rs-Prots, the gene expression patterns remained independent from the applied environmental conditions and relied only on the overall physiological state of the cell population (Figure 3) and characteristics of the promoter (Figure 2). Here, observed expression profiles reflect typical kinetics described previously for 4UASpTEF promoter (i.e., significant increase in expression in late exponential/early stationary growth phase) [38], confirming its pH-independence under sufficient OA. We postulate that the differences in the expression level between different OA variants, for both strains and under both pH levels, results from higher availability of transcriptional machinery elements necessary to proceed with transcription. Abundance of necessary transcription factors, polymerase elements, nucleotides and others were secured by higher rate of the nutrients consumption (Figure 1, Table 1) that could be obtained at a higher oxygen transfer capacity (i.e., kLa 110 h^−1^). Similar results were obtained by [22], where the Authors observed lower expression levels of a native lipase in *Y. lipolytica* cultured under oxygen deficiency. In addition, the critical role of OA for the expression level of heterologous genes in *Y. lipolytica* is further highlighted by the lack of significant impact of the medium pH on the heterologous gene transcription level (Figure 2).

The combination of intrinsic (metabolic load) and extrinsic (pH/OA) perturbations implemented in the present study allowed us to further develop the approach recently reported by [3]. In their work, the authors highlighted a positive correlation between the transcription and secretion of smaller β-galactosidase (55 kDa) and larger protein (120 kDa). Our experimental setup resulted in a much wider range of responses—from lack of correlation (e.g., SoA pH 7/low OA), negative correlation (any TlG), to significant positive correlation for secreted YFP under pH 7/high OA (r = 0.972; Figure 8). The range of biochemically different proteins and conditions tested allowed for additional conclusions. Primarily, the high OA, although of key importance for transcription and secretion, cannot completely over-balance the adverse impact of the burden imposed by overproduction of rs-Prots. In contrast, our recent study showed, that optimized thermal treatment (20 °C, 153 min) enables moderate improvement in rs-Prot synthesis in a metabolically overloaded strain [26], but it was not systematically studied in combination with differently burdened strains. Furthermore, we observed that even medium-sized proteins requesting intensive post-translational modifications impose a challenge to the translational-secretory machinery of *Y. lipolytica*, which results in a lack of correlation between transcription and translation. Finally, even for relatively “easy-to-produce” protein (YFP), limitation in OA impedes the flux through the protein synthesis and secretion machinery in producer host. 

## 4. Materials and Methods

### 4.1. Strains and Basic Culture Conditions

All *Y. lipolytica* strains used in this study, their genotype and phenotype characteristics, are listed in Table S1. A prototrophic derivative of Po1h strain was used as a negative control (i.e., not metabolically burdened strain) [4]. The low-burdened GGY251 strain (referred to as “LB” hereafter) express the gene encoding YFP. It was constructed by cloning the fusion of the YFP gene and the SP1 signal peptide [39], under the control of the 4UASpTEF promoter, as detailed elsewhere [4]. The highly burdened GGY178 strain (referred to as “HB” hereafter) co-express genes encoding two medium size proteins, namely high-disulphide bonded alpha-amylase SoA and highly glycosylated glucoamylase TlG. It was constructed by cloning the corresponding genes (with signal peptide SP1) under the promoter (4UASpTEF), as described previously [40].

Cultivation conditions for the strains subculturing or molecular biology protocols followed the standards described elsewhere [41,42]. Briefly, *Y. lipolytica* strains were grown in YNBG (Yeast Nitrogen Base supplemented with ammonium sulfate at 5 g L^−1^, glucose 20 g L^−1^) or YPD (Yeast extract, Peptone, Dextrose) media at 30 °C and 250 rpm. For solid media, agar 15 g L^−1^ was added.

### 4.2. Main Cultures and Implementation of Stress Factors

All batch cultures were conducted at a constant volume of 50 mL in Erlenmeyer shake-flasks of 100 ml or 1 L to obtain theoretical kLa coefficients of 28 h^−1^ and 110 h^−1^ as previously described (Gorczyca et al. 2020). The kLa coefficients were calculated as described elsewhere. Precultures were developed in 500 mL Erlenmeyer flasks containing 50 mL of YPD medium for 22 h at 28 °C and shaking of 250 rpm. Prior to inoculation of the final cultures, the biomass was separated from YPD medium by centrifugation (10 min, 5000 rcf) and resuspended in phosphate saline buffer. The main cultures were inoculated to reach an initial optical density at 600 nm of 0.5 (=0.66 g DCW L^−1^). The culture medium was composed as follows (g L^−1^): 1.7, YNB without amino acids and ammonium sulphate (BD Difco™, Sparks, NV, USA); 20, casein peptone (Biocorp Sp. z o.o., Warsaw, Poland); 20, glycerol. Media were buffered with 0.1 M phosphate buffer to maintain the desired pH (3 or 7) during the whole culture duration (72 h). Cultures were conducted in biological triplicates.

### 4.3. Gene Expression Analysis

RNA extraction and purification were conducted using the Bead-Beat Total RNA Mini kit (A&A Biotechnology, Gdynia, Poland) after mechanical disruption of the cells. For reverse transcription, a TranScriba Kit (A&A Biotechnology) was used with 5 ng of cDNA as a matrix. Real time quantitative PCR reaction (RT-qPCR) targeting YFP, SoA, TlG and actin encoding genes was conducted using RT PCR Mix SYBR^®^ B (A&A Biotechnology) and StepOnePlus Real-Time PCR System (Applied Biosystems, Foster City, CA, USA) apparatus. Actin-encoding gene (ACT1) was used as an internal calibrator for the normalisation of target gene expression. Cells from 0 h of the cultures were used as external calibrators. Data were processed according to the standard ΔΔCt method. Primers specific to actin and target genes are listed in Table S2.

### 4.4. Analytical Methods

Spectrophotometry and Fluorometry.

Cell growth was monitored through optical density measurement at 600 nm (OD600) using Thermo Scientific™ GENESYS™ 10S spectrophotometer (Madison, USA) and converted to gDCW L^−1^ based on experimentally defined equation y = 0.6103x + 0.3563 (R^2^ = 0.99, y = gDCW L^−1^, x = OD600). Quantification of YFP fluorescence in culture supernatants (outYFP) was performed using a Tecan Spark automatic plate reader (Tecan Group Ltd., Männedorf, Switzerland) with the following settings: λex and λem at 508 and 553 nm, respectively; bandwidth, 20 nm; gain, manual 67; mirror, automatic; number of flashes, 30; integration time, 40 μs; Z position, 20,000 μm [19]. Flat-bottom microtitre plates (Greigner 96 Flat Black) were used with sample size of 150 μl of culture supernatants. For each sampling time, values of endogenous fluorescence (FL) of the culture medium were subtracted from readouts of the culture supernatants (Figure S3). OutYFP was normalized to biomass (gDCW) to obtain specific YFP values (out sFU). All samples were analysed in technical triplicate, out of each of the three biological repetitions. 

#### 4.4.1. Flow Cytometry 

Intracellular YFP fluorescence (inYFP) was quantified using a BD Accuri C6 Flow Cytometer (BD Biosciences, San Jose, CA, USA) as described elsewhere. For each sample, 20,000 cells were analysed. Background noise (cell intrinsic fluorescence) was fixed at 20,000 fluorescence unit (FU) on FL1-A channel. This value encompasses the fluorescence level of the negative control cell (YFP non-producing). The flow cytometry dotplots FL1-A/FSC-A were analysed using CFlowPlus software (Accuri, BD Bioscience, San Jose, CA, USA). Gate Q1-UR and Q2-LR of the FLA1-FSC-A cytograms encompasses YFP producing and non-producing cells, respectively. 

#### 4.4.2. Enzymatic Assays

Alpha- and gluco-amylase activity were quantified by the microSIT method. One activity unit corresponds to the amount of enzyme that catalyses the hydrolysis of 1 mg of starch per 1 mL and 1 min at pH 5 and 40 °C, under applied experimental conditions. Specific extracellular activity (out sAU) was calculated by subtraction of background readouts (supernatant from cultures which enzymatic activity was stopped by the addition of HCl) and normalising the value to the biomass (gDCW). Extracellular amylolytic activity (AA) normalised per biomass is referred as out sAU (or separately: out sTIG and out sSoA). Measurements were conducted in technical duplicate, out of each biological triplicate.

#### 4.4.3. Chromatography

Concentration of carbon source was determined by isocratic RID-HPLC (Agilent 1100 series equipped with UV-visible and refractive index detector, Agilent Technologies, Santa Clara, CA, USA) using an Aminex HPX-87H ion-exclusion column (300 × 7.8 mm Bio-Rad, Hercules, CA, USA) with 5 mM H_2_SO_4_ as a mobile phase at a flow rate of 0.5 ml min^−1^ at 65 °C. 

#### 4.4.4. Microscopy

Microscopic slides were observed under magnification 1000×, using Axiovert 200M light fluorescent microscope (ZEISS, Oberkochen, Germany). The images were processed and archived using AxioVision software. Assessment of HB (strain GGY178) cells metabolic activity was conducted by staining cell with methylene blue for one minute. Metabolically active cell appeared colourless, while other remain stained. The results are expressed in percent, based on a manual count of over 5000 cells for each culture variant.

#### 4.4.5. Statistical Analysis

Initial data processing, graphical presentation of the data, and correlation analyses were conducted using Microsoft Excel and Data Analysis plugin. Analysis of correlation was conducted using regression test. The r-value represents the Pearson correlation coefficient and determines the linearity and trend of correlation between data sets (negative or positive). Significance of correlation is indicated by *p*-values. Statistical importance of the differences between compared sets of data was analysed using two-way analysis of variance (ANOVA) and Tukey’s multiple comparison tests using STATISTICA data analysis software system (StatSoft, Inc., Tulsa, OK, USA). The results were considered as statistically different at a *p*-value 0.05. The results were expressed as mean ± standard deviation (±SD) of the replicates, as indicated for a specific analysis.

## 5. Conclusions

In summary, this study provides an insight into the combined impact of intrinsic (metabolic load) and extrinsic (pH/OA) perturbations on cell growth parameters and rs-Prots synthesis in *Y. lipolytica*. We observed that highly burdened producer cells have higher demand for the substrate. Moreover, even when cell growth was compromised, the carbon source uptake rate remained unaffected. Our data highlighted that a low pH alone has minor impact on the cell growth, gene expression, and metabolic activity of the cell. However, it limits filamentation, which was previously shown to negatively impact on rs-Prots synthesis. On the other hand, a combination of low pH and limited OA exerts a strong detrimental impact on the producer cell in terms of cell growth, gene expression, and metabolic activity. Strikingly, we observed that these decreased performances result from an accumulation of less- or non-producing cell subpopulations rather than from the decreased synthetic capacity of a single cell, thereby limiting the overall amount of foreign protein that can be produced. These results contribute to a better understanding of the mechanisms of the yeast cells’ performance under exposure to intrinsic and extrinsic factors (alone or in combination), showing that the response may come from the induced heterogeneity of the population. In addition, current results have practical consequences for bioprocessing—if low OA cannot be avoided due to technical limitations (low kLa of bioreactor in operating conditions), the process should rather be conducted at higher pH, as a combination of these two factors at low value is highly detrimental for *Y. lipolytica* host cells, at least within the ranges studied here.

## Figures and Tables

**Table 1:** Parameters of flask cultures of *Y. lipolytica* strains overproducing YFP (GGY251—LB strain) and TlG+SoA (GGY178—HB strain) rs-Prots, under four culture variants, at 24 h of culturing. Growth rate and protein secretion were calculated within the exponential phase of growth (up to 24 h). Maximum biomass is given at selected time point, where the biomass accumulation peak was observed (refer to Figure 1). Numbers indicate mean values from biological triplicate ±SD. SD is given to a first significant digit after the comma. N.A.—not available; N.D.—not detected. a, b, c, d, e, f —homogenous groups not different at *p* < 0.05 based on ANOVA and Tukey’s multiple comparison tests. $—all are different between the LB and HB strains at *p* < 0.05 or less, except a.

pH and OA Conditions	Strain	Growth Rate Q_gDCW_ [g L^−1^ h^−1^]	Glycerol Consumption Rate Q_gly_ [g L^−1^ h^−1^] ^$^	Biomass Yield Y_x/s_ [g g^−1^] ^$^	Max. Biomass [g L^−1^]	Specific Secretion of rs-Prots [FU gDCW^−1^ or AU gDCW^−1^]	Total Secretion of Rs-Prots [FU or AU]
pH 3/kLa 28	LB	0.085 ± 0.002 ^d^	0.16 ± 0.01	0.54 ± 0.01	3.37 ± 0.03 ^f^	N.D.—irreversible degradation of rs-Prots under pH 3	
	HB	0.11 ± 0.01 ^d^	0.28 ± 0.01	0.39 ± 0.02	4.1 ± 0.1 ^f^		
	control	0.080 ± 0.002 ^d^	N.A.	N.A.	3.60 ± 0.01 ^f^	N.D.	N.D.
pH 3/kLa 110	LB	0.26 ± 0.02 ^b^	0.74 ± 0.01	0.36 ± 0.03	8.6 ± 0.5 ^bc^	N.D.—irreversible degradation of rs-Prots under pH 3	
	HB	0.275 ± 0.004 ^b^	0.65 ± 0.03	0.43 ± 0.02	8.4 ± 0.3 ^c^		
	control	0.28 ± 0.03 ^b^	N.A.	N.A.	7.3 ± 0.6 ^cd^	N.D.	N.D.
pH 7/kLa 28	LB	0.118 ± 0.002 ^d^	0.21 ± 0.01	0.57 ± 0.01	6.0 ± 0.2 ^e^	132 ± 27	459 ± 96
	HB	0.18 ± 0.01 ^c^	0.37 ± 0.02	0.50 ± 0.01	9.73 ± 0.03 ^b^	0.0029 ± 0.0004	0.015 ± 0.001
	control	0.113 ± 0.004 ^d^	N.A.	N.A.	6.1 ± 0.2 ^de^	N.D.	N.D.
pH 7/kLa 110	LB	0.46 ± 0.01 ^a^	0.83 ± 0.01 ^a^	0.55 ± 0.01	11.6 ± 0.1 ^a^	597 ± 83	6944 ± 1009
	HB	0.25 ± 0.02 ^b^	0.82 ± 0.01 ^a^	0.31 ± 0.02	7.5 ± 0.5 ^c^	0.0036 ± 0.0003	0.024 ± 0.002
	control	0.50 ± 0.02 ^a^	N.A.	N.A.	12.6 ± 0.5 ^a^	N.D.	N.D.

## Data Availability

All data accompanying this research are presented directly in the manuscript and Supplementary Materials.

## Supplementary Materials

The following supporting information can be downloaded at: https://www.mdpi.com/article/10.3390/ijms23073602/s1.
