# Supplementary material for: Synthesis of Secretory Proteins in Yarrowia lipolytica: Effect of Combined Stress Factors and Metabolic Load
Source: Int J Mol Sci. 2022 Mar 25;23(7):3602. doi: 10.3390/ijms23073602 (PMC8998316; doi:10.3390/ijms23073602)
Supplement: Supplementary file 1 [file ijms-23-03602-s001.zip › ijms-1635485-supplementary.pdf]

Figure S1

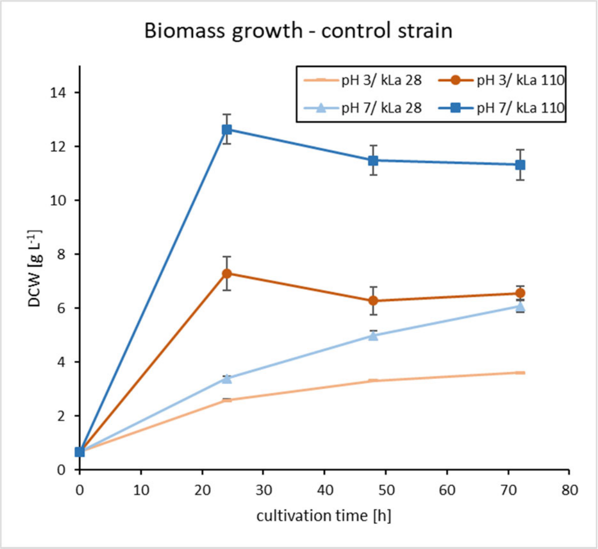

Figure S1. Biomass growth of *Y. lipolytica* prototrophic control strain under indicated conditions. Mean values of biological triplicate ±SD are shown. Colors and axes are explained in the provided legends.

Figure S2

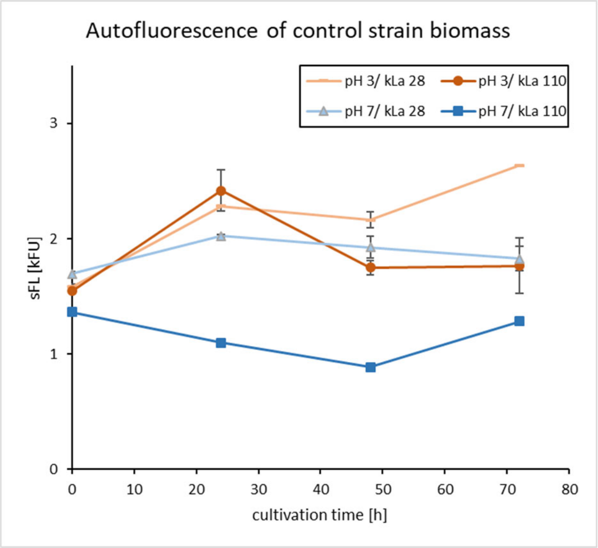

Figure S2. Time course of auto-fluorescence of *Y. lipolytica* prototrophic control strain under indicated conditions. The FL values were normalized per biomass. Mean values of biological triplicate ±SD are shown. Colors and axes are explained in the provided legends.

Figure S3

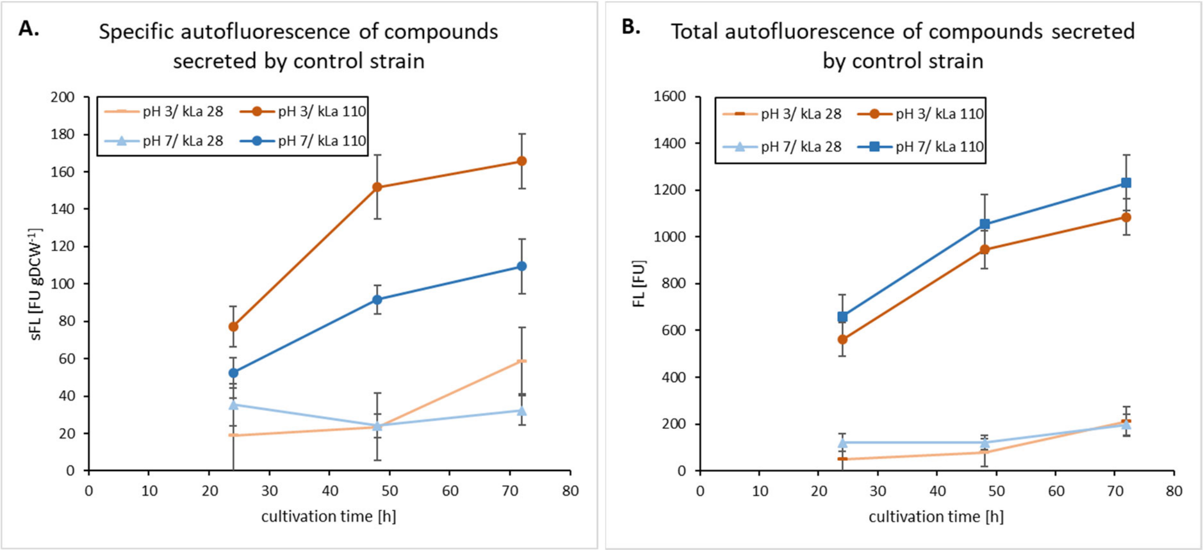

Figure S3. Time course of auto-fluorescence of compounds secreted by *Y. lipolytica* prototrophic control strain under indicated conditions. The specific FL values (A) were normalized per biomass. Mean values of biological triplicate ±SD are shown. Colors and axes are explained in the provided legends.

Figure S4

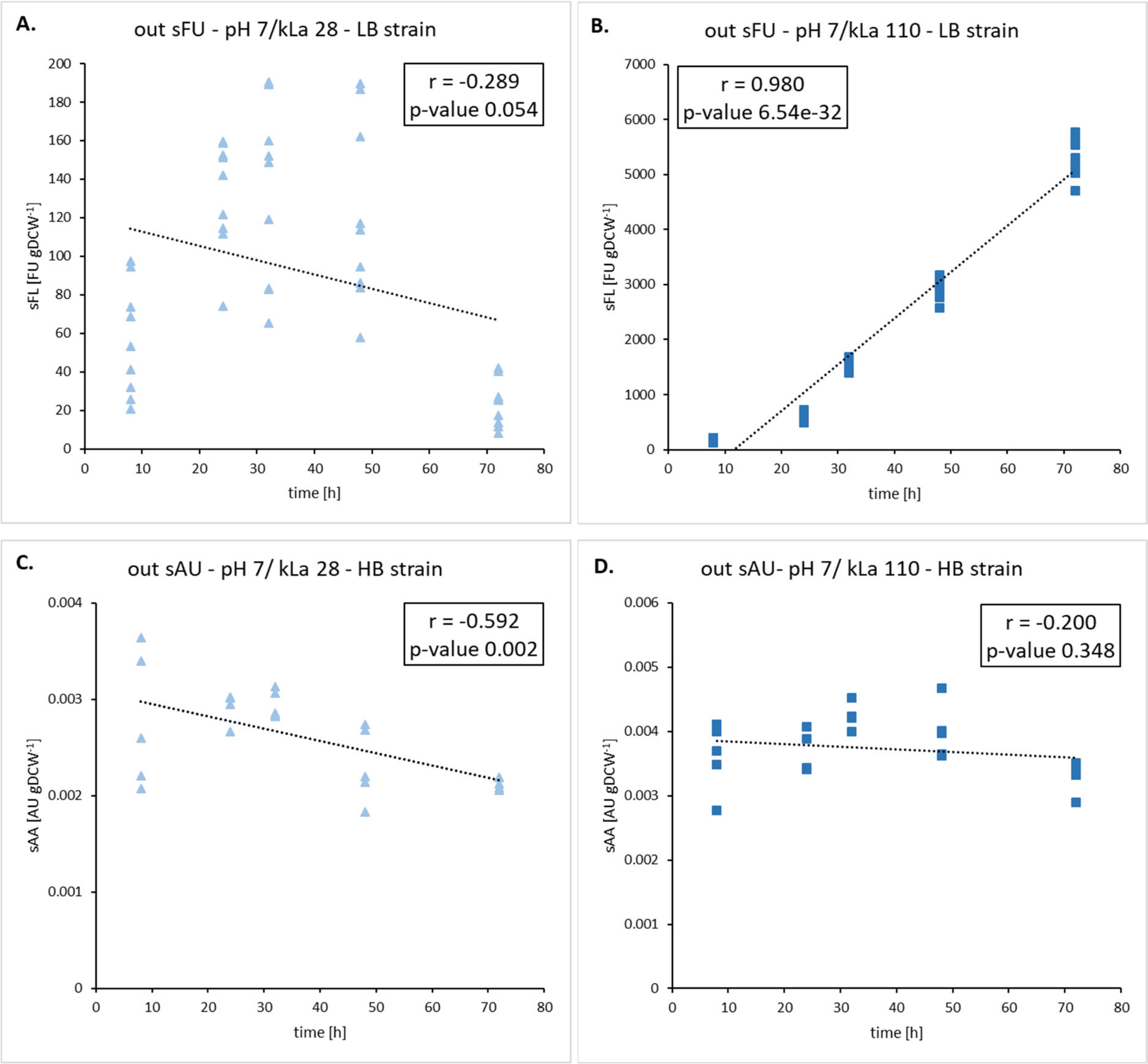

Figure S4. Correlation analysis for the extracellular FL (out sFU) (A, B) or AA (out sAU) (C, D) obtained under different OA conditions in *Y. lipolytica* strains cultures. Conditions are indicated on top of the sub-sections.

Table S1. Strains used in this study

Strain	Genotype	Phenotype
**Po1h**	*MatA, ura3-302, xpr2-322, axp1-2, leu2-270::LEU2*	ΔAEP, ΔAXP, suc+, ura-, leu+
**Po1h-Ura**	*MatA, ura3-302::URA3, xpr2-322, axp1-2, leu2-270::LEU2*	ΔAEP, ΔAXP, suc+, ura+, leu+
**GGY251**	*Po1h, URA3-p4UASpTEF-SP1-scYFP-tLip2*	Po1h, secretory YFP (LB strain)
**GGY178**	*Po1h,URA3-p4UASpTEF-TlG-tLip2-p4UASpTEF-SoA-tLip2*	Po1h, secretory TlG, secretory SoA (HB strain)

Table S2. Oligonucleotides used in this study

Analyzed gene	Primers sequence
**actin**	Forward 5' CGA GCG AAT GCA CAA GGA 3'
	Reverse 5' GCG GTG ATC TTG ACC TTG ATG 3'
**yellow fluorescent protein (YFP)**	Forward 5' CGA CCA CTA CCA GCA GAA CA 3'
	Reverse 5' CTT GTA CAG CTC GTC CAT GC 3'
**glucoamylase (TlG)**	Forward 5' TCT GAT GTT GTT TGG GAA TCT GA 3'
	Reverse 5' TGA TGT CTC CAC CAA GAG TTT ACT G 3'
**α-amylase (SoA)**	Forward 5' CTA CCG ACC ACG GAT TTG CT 3'
	Reverse 5' AGG ATC GGT AGA AGT GTC AAT CAC T 3'
